# Multilevel factors associated with virological suppression among adolescents and young people with prior non-suppression receiving intensive adherence counselling in East-Central Uganda

**DOI:** 10.1186/s12981-026-00901-5

**Published:** 2026-06-13

**Authors:** David Livingstone Ejalu, Juliana Namutundu, Sean Steven Puleh, Acen Joy, Ziadah Nankinga, Stella Immaculate Akech, Joanita Nangendo, Sabrina Bakeera-Kitaka, Achilles Katamba, Anne R. Katahoire, Joan Kalyango, Adithya Cattamanchi, Fred C. Semitala, Moses R. Kamya

**Affiliations:** 1 Clinical Epidemiology Unit, School of Medicine, College of Health Sciences, Makerere University, P.O Box 7072, Kampala, Uganda; 2 Faculty of Health Sciences, Uganda Martyrs University, Nkozi, Uganda; 3 School of Public Health, College of Health Sciences, Makerere University, Kampala, Uganda; 4 Infectious Diseases Research Collaboration, Kampala, Uganda; 5 Department of Medicine, University of California, Irvine, Irvine, CA USA; 6 Makerere University Joint AIDS Programme, Kampala, Uganda

**Keywords:** Virological suppression, Adherence support, Differentiated service delivery

## Abstract

**Background:**

Adolescents and young people living with HIV continue to experience disproportionately high rates of virological non-suppression despite scale-up of enhanced adherence interventions such as Intensive Adherence Counselling (IAC). While prior research has quantified post-IAC suppression outcomes, there remains limited empirical evidence regarding the multilevel factors that shape virological response in this population. This study investigated multilevel factors associated with viral load suppression among adolescents and young people (AYP) with prior non-suppression who were enrolled in IAC in East-Central Uganda.

**Methods:**

A facility-based analytical cross-sectional study was conducted using routinely collected programmatic data from 580 AYP aged 10–24 years with documented unsuppressed viral load who were enrolled in IAC across 32 health facilities. Eligible participants had a repeat viral load measurement following completion of the adherence intervention. Multivariable analysis was performed using modified Poisson regression with robust variance estimation to determine factors independently associated with virological suppression, accounting for clustering at the facility level. Adjusted prevalence ratios (aPRs) with corresponding 95% confidence intervals were reported.

**Results:**

The study population had a median age of 16.4 years (interquartile range [IQR]: 12.9–21.2), with females comprising 62.8% of participants. Overall, over half of the cohort achieved virological suppression following the intervention. Engagement in peer-led support was associated with a higher likelihood of suppression (aPR = 1.71, 95% CI: 1.36–2.15), as was prior disclosure of HIV status (aPR = 1.43, 95% CI: 1.02–2.11). Conversely, participants enrolled in differentiated antiretroviral therapy (ART) delivery models based on community drug distribution prior to IAC had lower suppression compared to those managed through facility-based individualized care (aPR = 0.51, 95% CI: 0.35–0.74).

**Conclusion:**

Virological outcomes among AYP receiving enhanced adherence support are shaped by a combination of individual and service delivery factors. Interventions that strengthen peer engagement and facilitate safe disclosure, alongside careful alignment of differentiated service delivery models with adherence needs, may improve treatment outcomes in this high-risk population.

## Introduction

Adolescents and young people (AYP) aged 10–24 years constitute a substantial proportion of the global HIV burden, representing a critical population within the epidemic. Globally, an estimated 6.75 million AYP are living with HIV, with approximately 84% residing in sub-Saharan Africa (SSA). This burden is further characterized by pronounced gender inequities, as adolescent girls and young women account for more than two-thirds of new infections in this age group [1, 2]. In Uganda, the epidemic similarly remains concentrated among AYP with an estimated 235,000 individuals living with HIV in 2024 [3, 4].

Despite high levels of enrolment of Adolescents and young people living with HIV (AYPLHIV) on antiretroviral therapy (ART), achieving sustained HIV viral load (VL) suppression remains a persistent public health challenge. Reported suppression rates among this population range from 44% to 88%, consistently falling short of the UNAIDS 95-95-95 targets [5, 6]. Compared with adults, AYPLHIV exhibit disproportionately higher levels of virological non-suppression, largely driven by suboptimal adherence to ART. Empirical evidence indicates that only approximately 20% of AYP achieve complete (100%) adherence within six months of treatment initiation, compared with nearly 40% among adults [7, 8]. National data from the Uganda Population-based HIV Impact Assessment (UPHIA) indicate that only 39.3% of children aged 0–14 years and 39.6% of young people aged 20–24 years are virally suppressed [9, 10].

To address virological non-suppression, the Uganda Ministry of Health (MOH) adopted Intensive Adherence Counselling (IAC) as a targeted intervention to improve treatment outcomes among clients with non-suppressed viral loads. IAC is indicated for individuals with a viral load ≥ 200 copies/mL after at least six months of ART or those experiencing virological rebound following prior suppression [11]. The intervention is premised on the assumption that poor adherence, rather than virological resistance, is the primary driver of non-suppression. It consists of a structured series of at least three monthly counselling sessions delivered by trained providers. Delivery of the intervention includes assessing adherence barriers, advising on the benefits of good adherence, assisting in identifying practical and actionable solutions, agreeing on a plan to address the barriers and arranging for follow-ups and refills. A repeat viral load test is conducted after completion of the initial sessions, and individuals with persistent non-suppression after six months of documented adherence are evaluated for transition to second-line ART [12–15].

Although IAC has been widely institutionalized within routine HIV care across Uganda, viral suppression among AYP remains suboptimal even after completion of the intervention. Evidence from Ugandan studies shows considerable variability in post-IAC suppression outcomes, ranging from as low as 23% among children and adolescents in multi-facility analyses to between 44% and 66% in adult clinic-based cohorts, and up to 86% in selected programmatic settings [16–19]. However, much of this literature has primarily focused on adult populations, or single-site evaluations, with limited disaggregation for AYP. Related studies conducted within Eastern Uganda, focused on adult populations without adolescent-specific analysis [20]. This leaves a gap in understanding of contextual and service delivery factors that influence suppression among non-suppressed AYP. Therefore, despite growing programmatic evidence on IAC effectiveness, there remains limited, context-specific and AYP-focused evidence particularly from East-Central Uganda on the factors associated with virological outcomes following IAC. This study addresses this gap by examining both individual- and health system-level factors associated with suppression among AYP enrolled in IAC in this high-burden setting.

## Methods

### Study design and setting

We implemented a retrospective facility-based analytical cross-sectional study utilizing routinely collected secondary data derived from both electronic medical record (EMR) systems and paper-based clinical documentation. The analytical framework was informed by the World Health Organization Multidimensional Adherence Model (WHO-MAM), which conceptualizes factors that influence treatment adherence across five domains: socio-economic, patient-related, therapy-related, condition-related, and health system factors [21]. Given the structure and completeness of routinely collected data, the present analysis was restricted to variables representing patient-level and health system domains, which were consistently documented across study sites.

The study was conducted in East-Central Uganda with a HIV prevalence of 4.5% and viral load suppression rate of 74.1% [22]. Data were obtained from 32 public-sector health facilities delivering ART services in Jinja City, Jinja District, Mayuge District, and Kamuli District that provide IAC in accordance with national HIV treatment guidelines [23]. Data was collected from 20 Health Centre IIIs (sub-county level facilities), nine Health Centre IVs (county level facilities), and three general hospitals (district level facilities) located within rural and peri-urban contexts to provide representative evidence. Prior research indicates that such settings are characterized by varrying structural and access-related constraints that may impede optimal treatment outcomes among AYPLHIV [24, 25]. This study setting was purposively chosen because the VL suppression among of 48.8 reported among AYP is the lowest reported in the country [9].

### Study population and sample size

The study population included virally non-suppressed AYP aged 10–24 years with documented virological non-suppression who were subsequently enrolled in Intensive Adherence Counselling. This population was selected due to persistent disparities in treatment outcomes relative to other age groups and its central relevance to achieving the third “95” target within the UNAIDS 95-95-95 targets [7, 26–28]. Sample size estimation was based on methods appropriate for assessing associations involving a binary outcome, with post-intervention viral load suppression as the primary endpoint. Parameter assumptions were informed by prior empirical studies: approximately 80% of adolescents were expected to achieve optimal adherence (≥ 95%), while 20% were anticipated to exhibit suboptimal adherence (< 95%). Corresponding viral suppression proportions were estimated at 69% among optimally adherent individuals and 50% among those with lower adherence [29–31]. Calculations assumed a 95% confidence level (Zα = 1.96) and 80% statistical power (Zβ = 0.84). To account for intra-facility correlation inherent in clustered service delivery data, a design effect of 2 was applied, consistent with established methodological guidance [32–34]. The resulting minimum sample size was 580 participants, providing adequate power to detect meaningful differences in virological outcomes across exposure groups.

### Sampling procedure

Eligible records were those of all AYP who were non-suppressed at the time of IAC initiation and had a documented follow-up viral load measurement after completing the adherence counselling sequence. Inclusion required completeness of key clinical and adherence-related variables within either EMR systems or paper-based patient records. Records lacking essential demographic or clinical information such as age, sex, or documented IAC initiation date were excluded to ensure analytical validity. A proportionate allocation strategy was applied to distribute the sample across participating facilities according to the relative number of eligible AYP enrolled in IAC at each site. Facilities with higher numbers contributed proportionally more records, thereby preserving representativeness across heterogeneous service delivery contexts. Within each facility, individual records were selected using simple random sampling procedures, minimizing systematic selection bias and enhancing the internal validity of the study.

### Data collection

Data extraction covered a five-year retrospective period and was conducted using multiple complementary sources, including EMR databases, standardized Ministry of Health registers, and individual patient clinical files. Information on adherence counselling attendance was obtained from structured IAC forms, while viral load outcomes were abstracted from the HMIS ACP 001 viral load register. Additional explanatory variables were retrieved from the HMIS ACP 003 HIV care card and corresponding EMR entries. A structured electronic data extraction tool was developed using KoboToolbox, with variable selection guided by the WHO-MAM framework to capture relevant patient-level and health system factors. Data collection was undertaken by five trained research assistants with prior experience in clinical data abstraction and digital data systems. They were trained on study protocols, ethical considerations, data confidentiality, and standardized abstraction procedures using tablet-based platforms. Prior to full-scale data collection, the extraction tool was piloted with data from 10 non-participating patient records to assess functionality and optimize the digital tool user experience for data collectors. Continuous supervision and periodic data quality checks were implemented throughout the data collection process to ensure adherence to protocol and minimize extraction errors.

### Study variables

The primary outcome was virological suppression, operationalized as a binary variable indicating whether an individual achieved viral suppression on the first viral load measurement following completion of IAC. In accordance with national guidelines, suppression thresholds were defined as < 1,000 copies/mL prior to July 2023 and < 200 copies/mL thereafter [11, 23]. Explanatory variables were categorized into patient-related and health system domains. Patient-level variables included demographic characteristics (age, sex, marital status, education level, and residence), clinical indicators (WHO stage, presence of opportunistic infections, ART duration, and clinical stability), and adherence-related measures (baseline adherence status, IAC compliance defined as ≥ 95% adherence score measured in pill counts in all three IAC sessions, and prior loss to follow-up). Psychosocial variables such as depression, anxiety, stress, fear, anger, and denial were also included where documented. Health system variables encompassed characteristics of service delivery and care processes, including the cadre of the adherence counsellor, ART delivery model prior to IAC initiation, drug refill practices, transfer-in status, ART regimen during IAC, history of tuberculosis preventive therapy, and availability of adherence support interventions such as peer support, disclosure counselling, treatment supporters, and household-level psychosocial support. Structural access factors, including distance to the health facility, were also examined.

### Data analysis

Data management and statistical analyses were conducted using STATA version 17.0 (StataCorp, College Station, Texas, USA). Initial data processing involved cleaning and validation in Microsoft Excel, including checks for completeness, consistency, and plausibility of key variables. Descriptive statistics were generated to summarize participant characteristics across patient-level and health system domains. Given that the prevalence of viral suppression exceeded 10%, associations between explanatory variables and the outcome were estimated using modified Poisson regression with robust standard errors, providing direct estimates of prevalence ratios. To account for clustering of observations within health facilities, robust variance estimation was applied at the facility level. Bivariate analyses were conducted to identify candidate variables for multivariable modelling, with a significance threshold of *p* < 0.05. Variables meeting this criterion were included in the initial multivariable model, and a backward elimination approach was employed to derive the most parsimonious model. Effect modification was assessed through inclusion of interaction terms between the primary exposure defined as adherence compliance (attendance of all IAC sessions with at least 95% adherence score) during IAC and other covariates. Statistical interaction was evaluated using likelihood ratio tests comparing nested models. Confounding was assessed by examining changes in effect estimates; variables resulting in a ≥ 10% change in the adjusted prevalence ratio were retained as confounders. Final model outputs were presented as adjusted prevalence ratios (aPRs) with corresponding 95% confidence intervals and p-values. Statistical significance was set at *p* < 0.05. Model adequacy was evaluated using the Hosmer-Lemeshow goodness-of-fit test.

## Results

### Study profile

**Fig. 1:**
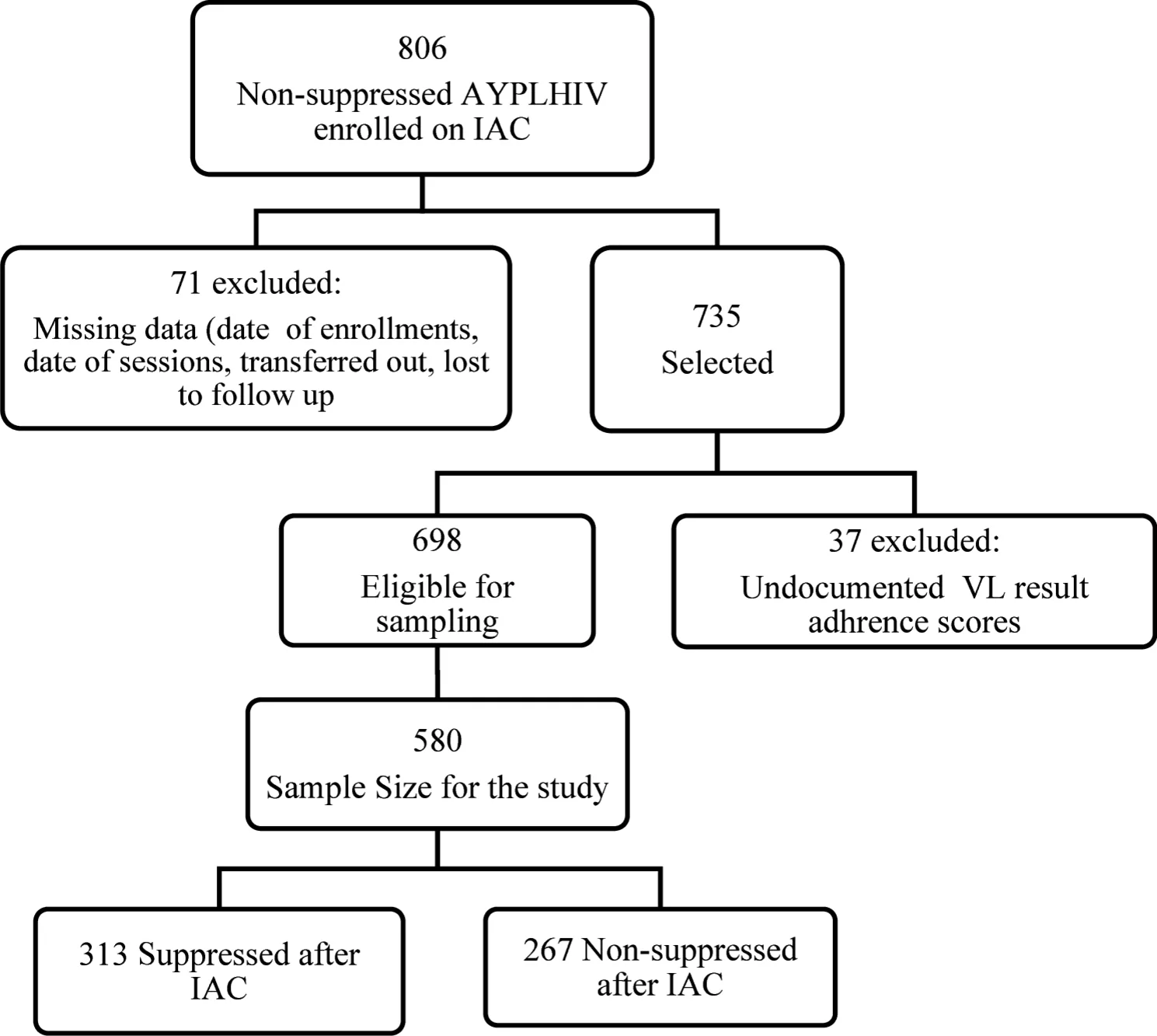
study flowchart

A total of 580 AYPLHIV underwent repeat viral load testing after completing the IAC period. Of these, 313 achieved VL suppression, while 267 remained virally non-suppressed. The flow chart summarizes the study population and the distribution of viral load outcomes following the IAC intervention (Fig. 1).

### Participants characteristics

Table 1 presents the socio-demographic characteristics of the 580 non-suppressed AYPLHIV who were enrolled in IAC. The median age of participants was 16.4 years (IQR: 12.9–21.2). Most participants were female (62.8%), while males accounted for 37.2%. The largest age category was 10–14 years (43.5%), followed by 20–24 years (32.9%) and 15–19 years (23.6%). The majority of participants resided in rural areas (74.8%) and had never been married (75.9%). Regarding education, 64.7% were currently in school, while 35.3% were out of school. The median duration on ART was 4 years (IQR: 2–6), with most participants having been on treatment for 1–4 years (62.9%). Nearly half of the participants (46.7%) received HIV care from Health Centre III facilities, followed by Health Centre IV (36.2%) and hospitals (17.1%).

**Table 1:** Demographic and clinical characteristics of AYPLHIV receiving IAC at selected health facilities in east central Uganda (*N* = 580)

Variable	Category	Frequency (*n*)	Percent (%)
Sex	Female	364	62.8
	Male	216	37.2
Age	10–14 years	252	43.5
	15–19 years	137	23.6
	20–24 years	191	32.9
Residence	Rural	434	74.8
	Urban	146	25.2
Marital status	Married	140	24.1
	Never married	440	75.9
Duration on ART	1–4 years	365	62.9
	5–9 years	131	22.6
	10 and above	84	14.5
Education status	Out of school	205	35.3
	In school	375	64.7
	More than 1	4	0.7
Employment status	Formal employment	7	1.2
	Not employed	198	34.1
	Student	375	64.7
Health facility level	H/C III	271	46.7
	H/C IV	210	36.2
	Hospital	99	17.1

### Factors associated with viral load suppression among non-suppressed AYPLHIV on IAC in East Central Uganda

**Table 2:** Multivariate analysis of factors associated with VL suppression among non-suppressed AYPLHIV on IAC in East Central Uganda

Variable	Category	Suppressed		Bivariate analysis		Multivariate analysis	
		No (*n* = 267)	Yes(*n* = 313)	uPR (95%CI)	*P* value	aPR (95%CI)	*P* value
Sex	Female	172(47.3%)	192(52.7)	1			
	Male	95(44.0)	121(56.0)	1.06(0.87–1.29)	0.553		
Age	10-14yrs	124(49.2)	128(50.8)	1		1	
	15-19yrs	58(42.3)	79(57.7)	1.14(0.93–1.39)	0.221	1.28(1.07–1.54)	0.007
	20-24yrs	85(44.5)	106(55.5)	1.09(0.84–1.42)	0.510	1.17(0.95–1.43)	0.144
Distance	> 5 km	139(52.6)	125(47.4)	1		1	
	< 5 km	128(40.5)	188(59.5)	1.26(1.08–1.46)	0.047	1.23(1.03–1.44)	0.022
IAC compliance	No	170(53.1)	150(46.9)	1		1	
	Yes	97(37.3)	163(62.7)	1.34(1.10–1.63)	0.004	1.37(1.16–1.62)	< 0.001
IAC provider	Nurse	56(75.7)	18(24.3)	1		1	
	counsellor	211(41.7)	295(58.3)	2.39(1.30–4.43)	0.005	2.12(1.24–3.61)	0.006
ART delivery model before IAC	FBIM	194(45.4)	233(54.6)	1		1	
	CDDP	28(75.7)	9(24.3)	0.45(0.30–0.67)	0.000	0.51(0.35–0.74)	< 0.001
	FBG	32(38.5)	51(61.5)	1.13(0.85–1.49)	0.414	1.12(0.88–1.39)	0.377
	FTDR	13(39.4)	20(60.6)	1.11(0.75–1.65)	0.604	1.31(0.86–2.01)	0.209
Peer Support	No	243(48.4)	259(51.6)	1		1	
	Yes	24(30.8)	54(69.2)	1.34(1.03–1.74)	0.027	1.71(1.36–2.15)	0.001
Disclosed status	No	31(63.3)	18(36.7)	1		1	
	Yes	236(44.4)	295(55.6)	1.51(1.02–2.23)	0.038	1.43(1.02–2.11)	0.042
*IAC compliance*peer support						0.60(0.41–0.88)	0.008

CDDP: Community Drug Distribution Points; FBIM: Facility-Based Individual Management; FBG: Facility Based Group; FTDR: Fast Track Drug Refill; uPR: unadjusted prevalence ratio; aPR: adjusted prevalence ratio

*IAC compliance*peer support is an interaction term

In the adjusted multivariate model shown in Table 2, AYPLHIV aged 15–19 years were significantly more likely to achieve viral load suppression compared to those aged 10–14 years (aPR = 1.28, 95% CI: 1.07–1.54, *p* = 0.007). Participants who lived within 5 km of the health facility had a 23% increased likelihood of suppression than those residing beyond 5 km (aPR = 1.23, 95% CI: 1.03–1.44, *p* = 0.022). Receiving IAC from a counselor rather than a nurse was associated with more than double the likelihood of viral suppression (aPR = 2.12, 95% CI: 1.24–3.61, *p* = 0.006). Participants enrolled in the Community Drug Distribution Points (CDDP) model before IAC were significantly less likely to achieve suppression compared to those in the Facility-Based Individual Management (FBIM) model (aPR = 0.51, 95% CI: 0.35–0.74, *p* = 0.001). AYPLHIV who reported receiving peer support had a 71% higher likelihood of suppression than those without peer support (aPR = 1.71, 95% CI: 1.36–2.15, *p* = 0.001). Disclosure of HIV status was also significantly associated with higher suppression outcomes (aPR = 1.43, 95% CI: 1.02–2.11, *p* = 0.042). A significant interaction was observed between IAC compliance and peer support (aPR = 0.60, 95% CI: 0.41–0.88, *p* = 0.008).

## Discussion

This study assessed VL suppression in previously unsuppressed AYPLHIV recruited in IAC in East-Central Uganda and determined suppression-associated factors. Overall, 54% achieved VL suppression after IAC. This IAC performance among this population and setting is well behind both the 85% recorded among adults getting IAC in Uganda and the national target of 70% [35]. This disparity reflects age-specific adherence vulnerabilities, such as structural and psychosocial barriers, including financial limitations and long travel times to facilities that limit AYP’s capacity to get routine IAC services as effectively as possible. The observed estimate is comparable to the pooled estimate of 51.2% from a recent meta-analysis of enhanced adherence counselling interventions in Africa [36] and is consistent with earlier Ugandan studies that reported suppression levels between 23% and 51% [18, 37, 38]. These results imply that although IAC increases viral suppression, the multi-level adherence hurdles typical of AYPLHIV may not be adequately addressed by its present delivery method. In light of IAC, more AYP-responsive delivery strategies are required to address specific adherence barriers.

We further examined factors associated with VL suppression and found that AYPLHIV between the ages of 15 and 19 were 28% more likely to achieve VL suppression compared to those aged 10–14 years. According to Dinaj-Koci et al. (2019) and Mayeye et al. (2019), age-related variations in VL suppression point to a behavioural and developmental process that underlies adherence, likely reflecting increased autonomy, cognitive maturity, and self-regulatory competence that enable consistent medication-taking [39, 40]. Younger adolescents, on the other hand, continue to be structurally dependent on their caregivers; as a result, adherence is dependent on elements such as home stability, disclosure dynamics, and caregiver reliability, which introduce variability and may cause treatment interruptions [41]. These mechanisms are further reinforced by geographic proximity. AYPLHIV living within 5 km of a facility was associated with a 23% higher likelihood of suppression. This is probably mediated by lower transportation costs, better clinic attendance, and continuous exposure to IAC [42, 43]. These results highlight the significance of decentralising IAC delivery through differentiated service delivery models (DSD) models to reduce structural hurdles, especially for younger and geographically disadvantaged AYP.

Full compliance with IAC was independently associated with VL suppression. There was a 37% higher likelihood of those attending all sessions with at least 95% adherence score to suppress. These results support the dose-response relationship whereby repeated counselling exposures reinforce adherence behaviors. Evidence from Kenya and Cameroon demonstrates substantially higher odds of suppression among AYP with optimal IAC attendance [44, 45]. In addition, peer support showed a 71% higher likelihood of improving VL suppression. Peer relationships are known to work in a number of ways, such as lowering internalized stigma, boosting self-efficacy, and offering contextually appropriate adherence techniques based on common lived experiences [46–48]. Evidence from Zimbabwe and Uganda supports the effectiveness of peer-led AYP interventions in improving suppression outcomes [49, 50]. Importantly, we observed sub-additive interaction between peer support and IAC compliance. Our results indicate that the effect of compliance is modified by peer support, such that the combined effect of both does not equal the simple multiplicative sum of their individual effects. This suggests that both interventions maybe working on comparable behavioral pathways such that their combined effect does not produce proportionate benefits. The findings imply that rather than programs layering interventions, optimizing their integration and targeting distinct behavioral factors may yield greater VL outcomes. The facts about these interaction terms calls for further research.

We further noted that counsellor-delivered IAC was associated with a two-fold higher likelihood of suppression compared to nurse-led delivery. This maybe because counsellors are trained in behavioral counselling techniques and are perceived to offer greater privacy and more non-judgmental communication, which increases credibility and trust compared with routine nurse encounters. Direct evidence comparing counselor-led versus nurse-led IAC on viral suppression is lacking. Multiple programmatic and research reports describe IAC delivered by multidisciplinary teams or by mixed cadres without reporting outcomes stratified by who delivered the counseling [13, 19, 36, 51–53]. Although there is limited direct evidence comparing the effectiveness of nurse-led and professional counsellor-led counselling, specifically among virally non-suppressed HIV-positive AYP. This finding suggests that counsellors are better equipped to provide targeted adherence support compared to nurses who may have broader clinical responsibilities.

In contrast, prior enrolment in CDDP was associated with a 50% lower likelihood of VL suppression relative to FBIM. This finding reflects a disruption mechanism whereby transitioning clients from community-based models to facility-based IAC negates the structural advantages of CDDP, including reduced transport burden, enhanced privacy, and minimized stigma [54, 55]. This finding shows that the misalignment between IAC delivery and DSD principles inadvertently disadvantages individuals previously stabilized in community-based care. Additionally, disclosure was positively associated with a 43% higher likelihood of suppression. This is explained by the increased social support, improved communication with caregivers, and reduced psychological distress, all of which facilitate adherence [56–58]. Therefore, innovative approaches for disclosure and delivery IAC through the various DSD models are required to sustain the benefits of the models in shaping adherence outcomes.

This study has several strengths, including the use of multi-facility routine data from 32 public health facilities across diverse rural and peri-urban settings, enhancing external validity. The focus on a high-risk, non-suppressed AYPLHIV population and the application of clustered Poisson regression strengthen both the relevance and methodological robustness of the findings. However, the study had some limitations to be considered. The reliance on routine data could potentially introduce information bias, particularly in adherence measurement based on pill counts. Furthermore, the restriction to patient and health system level variables, guided by WHO-MAM, may have excluded broader structural and community-level factors, potentially underestimating the complexity of factors that influence post IAC viral suppression.

## Conclusion

This study’s finding reveals the critical role of IAC in improving VL suppression among non-suppressed AYPLHIV. Several key factors were significantly associated with improved VL suppression, including proximity to health facilities, receipt of peer support, HIV status disclosure, and being counseled by professional counselors rather than general nurses. These findings suggest that both individual and system elements play a crucial role in influencing IAC outcomes. Importantly, AYPLHIV who transitioned from community-based models and multi-months’ refill to facility-based models and monthly refills, respectively, during IAC were less likely to achieve suppression, indicating the need to adapt IAC to fit within differentiated service delivery models to maintain accessibility and continuity of care. We recommend integrating age-specific support, enhancing access through high impact community-based platforms, and leveraging peer-led strategies to reinforce motivation and reduce stigma. Additionally, efforts to strengthen the counseling workforce and ensure psychological support, especially through the recruitment of trained counselors at health centre III level facilities. Further research on implementation strategies that focus on optimizing IAC within differentiated care models and incorporating AYP-responsive components is needed to enhance long-term treatment outcomes.

## Data Availability

No datasets were generated or analysed during the current study.

## Abbreviations

ART: Antiretroviral therapy
IAC: Intensive adherence counseling
VL: Viral load
AYPLHIV: Adolescents and young people living with HIV
CDDP: Community drug distribution points
FBIM: Facility-based individual management
